# The Effect of the Hospital Readmission Reduction Program on the Duration of Observation Stays: Using Regression Discontinuity to Estimate Causal Effects

**DOI:** 10.5334/egems.197

**Published:** 2017-12-15

**Authors:** Jordan Albritton, Thomas Belnap, Lucy Savitz

**Affiliations:** 1 Intermountain Healthcare, US; 2 Kaiser Permanente Center for Health Research, US

**Keywords:** Health Policy, Readmissions, Observation stays, Regression Discontinuity, Quasi-experimental, Hospitals

## Abstract

**Research Objective:**

Determine whether hospitals are increasing the duration of observation stays following index admission for heart failure to avoid potential payment penalties from the Hospital Readmission Reduction Program.

**Study Design:**

The Hospital Readmission Reduction Program applies a 30-day cutoff after which readmissions are no longer penalized. Given this seemingly arbitrary cutoff, we use regression discontinuity design, a quasi-experimental research design that can be used to make causal inferences.

**Population Studied:**

The High Value Healthcare Collaborative includes member healthcare systems covering 57% of the nation’s hospital referral regions. We used Medicare claims data including all patients residing within these regions. The study included patients with index admissions for heart failure from January 1, 2012 to June 30, 2015 and a subsequent observation stay within 60 days. We excluded hospitals with fewer than 25 heart failure readmissions in a year or fewer than 5 observation stays in a year and patients with subsequent observation stays at a different hospital.

**Principal Findings:**

Overall, there was no discontinuity at the 30-day cutoff in the duration of observation stays, the percent of observation stays over 12 hours, or the percent of observation stays over 24 hours. In the sub-analysis, the discontinuity was significant for non-penalized.

**Conclusion:**

The findings reveal evidence that the HRRP has resulted in an increase in the duration of observation stays for some non-penalized hospitals.

## Introduction

Due to their ability to eliminate bias and estimate causal treatment effects, randomized controlled trials (RCTs) are considered the gold standard in most published hierarchies or levels of evidence [1,2]. However, despite offering high quality evidence, RCTs are not without their limitations. First, clinicians may have difficulty applying evidence from carefully controlled RCT environments to the complex real-world settings [3]. Second, and perhaps more importantly, many interventions or research questions present one or more challenges that make an RCT approach impractical. Examples include organizational studies, policy analyses, quality improvement, and studies where it would be unethical to withhold a treatment or intervention from patients. RCTs also require a significant resource investment, making them difficult to apply in traditional care settings.

The challenges associated with RCTs make observational studies common. However, in most cases, these studies provide a lower quality of evidence and are rife with potential bias [2,3]. Another alternative is the regression discontinuity (RD) design, a quasi-experimental research design that can offer the robustness of an RCT [4]. Although the RD design only works in certain situations, RD can be applied using observational data, requiring a fraction of the investment of an RCT. However, the RD design is rarely used in health services research [5]. Given the increasing abundance of data available in electronic health records (EHR) and medical claims, there is a growing opportunity to use RD to generate evidence in health care, including robust estimations of causal effects. As an example, we demonstrate the use of RD to estimate the impact of the Hospital Readmission Reduction Program (HRRP) on the duration of observation stays for heart failure (HF).

### Hospital Readmission Reduction Program

Established as part of the Affordable Care Act, the HRRP reduces Medicare prospective payments (up to 3 percent) for hospitals with more than expected 30-day readmissions [6]. Penalties are based on the excess readmission ratio (ERR), a measure that adjusts for risk and compares hospital readmission rates to the national average. Hospitals with an ERR greater than 1.0 across a 3-year measurement period receive a payment penalty [7]. For example, data from July 1, 2012, to June 30, 2015, are used to determine penalties for fiscal year 2017. The first penalties were levied in 2012 for HF, myocardial infarction, and pneumonia – additional conditions were added later [7].

Unsurprisingly, hospital leaders have also expressed concern about the HRRP, particularly regarding the size of penalties, the risk adjustment process, and hospitals’ inability to significantly impact patient adherence [8]. Regardless of improvements, due to the structure of the program, approximately half of all hospitals will always face a penalty. Critics also question the effectiveness and accuracy of the risk adjustment involved in the calculation of the ERR [9,10]. Despite the risk adjustment, hospitals serving a large number of low-income patients and/or dual-eligible patients are much more likely to receive penalties [11,12]. Additionally, many question the ability of hospitals to reduce readmissions throughout the seemingly arbitrary 30-day time frame. Readmission rates have been shown to be largely unrelated to care quality [13,14]. Indeed, research shows that the greatest determinants of readmission are factors that are largely out of hospital control, especially after 7 days [15,16,17].

### Observation Stays

Given the challenges hospitals face in reducing readmissions and the financial pressure of the HRRP, there is concern that hospitals may seek other ways to avoid potential payment reduction penalties. Some have suggested that the HRRP creates a financial incentive to reduce even necessary readmissions, which could result in increased mortality [12]. Alternatively, hospitals could place patients on observation status, a care classification that does not count towards readmission penalties [18,19]. The latter has been the focus of a growing debate. Although studies have shown that overall rates of readmission for HF and other conditions have been declining for more than a decade, observation stay rates have been rising in this time period as well [20,21,22,23,24]. Despite these trends, other researchers have shown that the increase in observation stays is too small to account for the decrease in readmissions [20,25].

Much of the work focused on observation stays uses observational, nonexperimental research designs that limit the ability to draw firm causal links. Additionally, while the decrease in readmissions exceeds the increase in observation stays, the effect on observation stays may not be insignificant. It is also possible that some hospitals work to shift patients to observation status more than others. In fact, hospital characteristics, such as ownership, size, and location, are among the most significant predictors of both the frequency and duration of observation stays among Medicare beneficiaries [26]. Failing to account for heterogeneity could bias results towards null findings. The duration of observation stays has also been increasing over the past several years, despite the fact that observation stays are intended to be no longer than 48 hours [18,22,27]. The HRRP could incentivize hospitals to keep patients on observation status longer in an effort to avoid readmissions.

### Heart Failure

We focus on HF for several key reasons. First, HF affects 17 percent of the Medicare population over the age of 65 and the prevalence is increasing [28,29,30]. Second, HF is one of the leading causes of 30-day readmissions as nearly a quarter of all hospital stays for HF result in a readmission within 30-days [21]. HF accounted for 7.3 percent of all 30-day readmissions among Medicare beneficiaries in 2011, more than any other condition [31]. Finally, HF was one of the first three conditions targeted by the HRRP, meaning that hospitals have had years to work on finding ways to reduce readmissions.

### Summary

In this article, we use RD to estimate the impact of the HRRP on the duration of observation stays following hospitalization for HF. We hypothesize that the HRRP increases the average duration of observation stays for HF, particularly near the near day 30, where the potential to avoid a 30-day readmission increases. Also, we expect greater evidence of an impact of the HRRP among non-penalized hospitals (ERR ≤ 1) because they may have a more comprehensive strategy to reduce readmissions. Thus, we conduct a sub-group analysis comparing penalized and non-penalized hospitals.

## Methods

### Data

This work was conducted through a partnership with the High Value Healthcare Collaborative (HVHC), a network of 12 learning health care systems across the United States. We included all Medicare patients in hospital referral regions (HRR) represented by HVHC members, 57 percent of the nation’s HRRs. We limited the analysis to patients with an index admission for heart failure from July 1, 2012 to June 30, 2015. The analysis was also limited to patients that had an observation stay between 6 and 55 days after discharge from the hospital. We excluded hospitals with fewer than 25 readmissions in a calendar year as they are not affected by the HRRP. We also excluded hospitals with fewer than 5 observation stays because we were interested in hospitals using observation stays frequently enough to potentially impact their ERR scores. Lastly, we excluded observation stays that occurred at a different hospital than the index admission. The new hospital would not necessarily be incentivized to use observation status to avoid readmissions. After exclusions, the sample consisted of 19,815 observation stays across the 3-year period. Overall, the dataset included 419 hospitals.

### Approach

RD can be used to make causal inferences, even when randomization is not possible or practical [5,32]. Compared to other quasi-experimental or observational research designs, RD requires fewer, simpler assumptions [4,32]. The primary requirement of RD is an assignment variable with a seemingly arbitrary cutoff used to determine treatment [32]. If there is little or no difference between subjects on either side of the cutoff, then these subjects can be seen as essentially randomized into treatment and control groups. Trends are plotted on either side of the cutoff; the discontinuity in the trends is an unbiased estimate of the treatment effect [4]. The HRRP applies a 30-day cutoff after which readmissions no longer contribute to reimbursement penalties [12]. In reality, patients readmitted on the 30^th^ day following an index admission are likely not very different from patients readmitted on the 31^st^ day. As a result, the HRRP is a prime example of a policy that can be evaluated using RD.

While several statistical programs (e.g., Stata, R) include packages for conducting RD analysis, the analysis can also be conducted using simple regression equations. Equation 1 shows the general linear form of the RD equation. In Equation 1, *β*_1_ is the slope of the line before the cutoff, *β*_2_ is the slope of the line after the cutoff, and *τ* is the treatment effect. X is the assignment variable, c is the treatment cutoff value, and Y is the outcome variable. D is equal to 1 when X ≥ c and D is equal to 0 when X < c. In the absence of a cutoff-based treatment there is no discontinuity and *τ* will equal 0. Control variables can be included in the analysis, but are not typically necessary to achieve unbiased results [4]. Higher-order equations are also possible as shown in Equation 2, the general quadratic form of the RD equation. Although including higher-order terms can reduce estimate precision, improper functional form produces biased results. Thus, when using parametric equations, Lee and Lemiuex recommend comparing the fit of the linear model to higher-order models to identify the specification that best fits the underlying data [4]. Non-parametric equations represent an alternative approach utilized by many statistical packages [33].

Equation 1 Y = \,\,a\,\, + \,\,\tau D\,\, + \,\,{\beta _1}\left( {X - \,\,c} \right)\,\, + \,{\beta _2}D\left( {X - \,\,c} \right)\,\, + \,\,\varepsilon

Equation 2 Y = \,\,a\,\, + \,\,\tau D\,\, + {\beta _1}\left( {X - \,\,c} \right) + {\beta _2}{\left( {X - \,\,c} \right)^2} + \,\,{\beta _3}D\left( {X - \,\,c} \right) + \,{\beta _4}D{\left( {X - \,\,c} \right)^2} + \,\,\varepsilon

In addition to functional form, the bandwidth of data used for the analysis can also impact RD results. Because RD is only valid for subjects with assignment variable scores near the cutoff value, the inclusion of data far from the cutoff value could bias results [34]. On the other hand, using a narrow bandwidth reduces the precision of the estimate of the treatment effect. Thus, the optimal bandwidth should minimize the risk of bias while providing precise estimates. Imbens and Kalyanaraman offer one way to empirically estimate the optimal bandwidth (the IK bandwidth) by minimizing the mean squared error [34]. Still, due to questions about the optimal bandwidth and ideal functional form, most researchers recommend using multiple RD models – results that are stable across multiple plausible model specifications will be seen as more reliable than results that are only significant with a particular set of conditions [4].

In addition to requiring a seemingly arbitrary cutoff, RD analysis typically involves testing two key assumptions. First, research subjects must be unable to self-sort [4]. Researchers test for self-sorting by analyzing the density of the data across the cutoff. A jump in the density from one side of the cutoff to the other suggests that some research subjects successfully altered their assignment variable value to fall in the treatment or control group. Self-sorting is unlikely in this analysis because the HRRP affects payments to hospitals, but potential penalties are based primarily on patient behavior and outcomes. Patients are unlikely to choose to present at a hospital on day 31 instead of day 30 in order to prevent the hospital from being penalized. We confirmed that there was no evidence of self-sorting by examining the density in the total number of observation stays and readmissions by days from discharge. The second key assumption is that there are no discontinuities in baseline covariates at the cutoff. Indeed, RD’s greatest strength is its ability to balance observed covariates on either side of the cutoff [5]. At the very least, violation of this assumption indicates that research subjects were able to manipulate the assignment variable to some degree. Our data included the average age of subjects; we found no evidence of a discontinuity. Overall, we found no evidence of a violation of these two key assumptions.

### Analyses

We used the duration of observation stays in hours as our primary outcome measure. Secondary outcome measures included the percent of observation stays that were greater than 12 hours and 24 hours in duration. We conducted RD analysis with the three outcome variables using days from discharge as our assignment variable. We set the cutoff value to 30.5 days, corresponding to the HRRP. The analyses were conducted using linear regression analysis in Stata; we used the ‘sureg’ command for seemingly unrelated regressions to improve the efficiency of the standard errors. We conducted nonparametric RD in R using the ‘rdrobust’ and ‘rdd’ packages. We set alpha to 0.05 for hypothesis tests and applied the Benjamini-Hochberg (BH) procedure to correct the p-value limit for multiple tests [35].

Limiting the analysis to patients with an observation stay between 6 and 55 days after the index admission largely eliminated nonlinearities in the data. Likelihood ratio (LR) tests confirmed that linear models fit the data better than higher-order models for each outcome variable [4]. Thus, we used a linear model with the full bandwidth for the analysis. To test the consistency of any significant results, we also estimated three additional models: 1) linear models with the IK bandwidth; 2) nonparametric models with the full bandwidth; and 3) nonparametric models with the IK bandwidth. We used a rectangular kernel for the parametric models because it has a more straightforward interpretation. We used a triangular kernel for nonparametric models—the triangular kernel weights data based on proximity to the cutoff. We repeated the process for the sub-group analysis, separately estimating the effect of the policy for penalized and non-penalized hospitals. This project was approved the Institutional Review Board at Dartmouth College.

## Results

**Figure 1:**
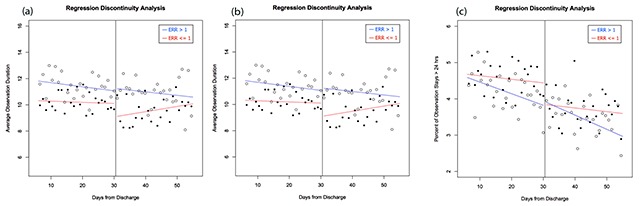
Results from Regression Discontinuity Analyses. Legend Blue regression lines, “o” plot points: Penalized hospitals (ERR > 1). Red regression lines, “•” plot points: Non-penalized hospitals (ERR ≤ 1). Results from regression discontinuity analysis using the average duration of observation stays in hours as the outcome variable.Results from the regression discontinuity analysis using the percent of observation stays over 12 hours as the outcome variable.Results from the regression discontinuity analysis using the percent of observation stays over 24 hours as the outcome variable.

The results from the main RD analyses are shown in Table 1. We present the magnitude, p-value, and 95 percent confident interval of the discontinuity (i.e., the treatment effect, *τ*, from Equation 1) from linear models using the full bandwidth. There were no statistically significant discontinuities in the overall analyses (models 1–3). The sub-group analysis also showed insignificant results for hospitals receiving a penalty due to the HRRP (models 4–6). However, the discontinuities for non-penalized hospitals (models 7–9) were statistically significant, suggesting that the HRRP increases the duration of observation stays at non-penalized hospitals. The results of the sub-analysis are also presented graphically in Figure 1. For non-penalized hospitals, the discontinuities from day 30 to day 31 correspond to a reduction in the average duration of observation stays by 0.996 hours (95 percent CI –1.850 to –0.143, *P* = 0.022), a reduction in the percentage of observation stays longer than 12 hours of 0.752 percentage points (95 percent CI –1.310 to –0.193, *P* = 0.008), and a reduction in the percentage of observation stays longer than 24 hours of 0.0591 percentage points (95 percent CI –1.087 to –0.095, *P* = 0.019). As shown in Table 1, the discontinuities in the duration of observation stays and the percent of observation stays greater than 12 hours at non-penalized hospitals remained significant after BH correction for multiple tests (n = 6); the discontinuity in the percent of observation stays greater than 24 hours was just over the adjusted cutoff (*P* = 0.019 vs. 0.017).

**Table 1:** Results from regression discontinuity analysis.

	coef (t)	p-value	95% CI(LL, UL)	BHp-value limit

Effect of HRRP on all hospitals				
1 – Average duration of observation stays	0.465	0.149	(–0.166, 1.095)	0.067
2 – Percent of observation stays > 12 hrs	0.366	0.159	(–0.143, 0.876)	0.083
3 – Percent of observation stays > 24 hrs	0.236	0.208	(–0.131, 0.604)	0.100
Effect of HRRP on penalized hospitals (ERR > 1)				
4 – Average duration of observation stays	–0.189	0.724	(–1.237, 0.860)	0.033
5 – Percent of observation stays > 12 hrs	–0.148	0.678	(–0.847, 0.551)	0.042
6 – Percent of observation stays > 24 hrs	–0.116	0.666	(–0.643, 0.411)	0.050
Effect of HRRP on non-penalized hospitals (ERR ≤ 1)				
7 – Average duration of observation stays	0.996	0.022*	(0.143, 1.850)	0.025
8 – Percent of observation stays > 12 hrs	0.752	0.008*	(0.193, 1.310)	0.008
9 – Percent of observation stays > 24 hrs	0.591	0.019	(0.095, 1.087)	0.017

*Notes*: * indicates significance using the BH corrected p-value with α = 0.05. The BH p-value limit is equal to [α/n * r] where n is the number of tests and r is the p-value significance rank from smallest to largest. For the overall analysis n = 3; for the sub-analysis, n = 6.

## Discussion

These findings show that the HRRP has had an effect on the duration of observation stays for some hospitals. The overall results show that the HRRP has had no impact on the duration of observation stays following index admissions for HF, but the sub-group analysis provides additional granularity. Although there was no effect among penalized hospitals, there was a moderate effect among non-penalized hospitals. Specifically, the results show that the HRRP increases the duration of observation stays at non-penalized hospitals, particularly near the 30-day cutoff.

This study suggests that the use or extension of observation status may be one strategy hospitals adopt to help reduce readmissions for HF. Observation status is certainly appropriate under the right circumstances, but the use of observation status solely to avoid penalties could adversely affect the cost or quality of patient care. Whereas the cost for inpatient care is capped at the inpatient deductible, hospitals bill observation care as an outpatient service with a 20 percent coinsurance. As a result, longer observation stays are associated with higher costs; observation stays greater than 24 hours are significantly higher costs than a corresponding inpatient stay [36]. Additionally, evidence shows that the duration of observation stays has increased over the past decade [23]. This has important implications as observation care does not count towards the 3-day minimum inpatient stay required for Medicare to cover care in skilled nursing facilities (SNF) [22]. As a result, patients on observation status could face higher costs for similar care received in a hospital bed and then find themselves without responsible for the cost of SNF care. The two-Midnight Rule would have addressed this concern by reclassifying observation stays that crossed two midnights as inpatient stays, but the policy was eventually eliminated [37]. Given this new evidence, the increasing utilization and duration of observation status, and the potential impact on patients, we believe the policy change may be necessary to ensure better alignment between hospital incentives and what’s best for the patient. Research on the HRRP and other policies aimed at influencing hospital behavior should remain a high priority.

This analysis has a few key limitations. First, the results are not significant in alternative nonparametric models and models using a shorter bandwidth of data. Although LR tests show that the linear model fits the data well, robust results that remain significant across alternative model specifications are more reliable and resistant to bias [4]. Second, the sub-group analysis suggests that there may be heterogeneous effects; we explored one moderating factor, but other factors likely influence how hospitals respond to policies and incentives. Third, we can only interpret the results for patients near the 30-day cutoff. The impact of the HRRP could be larger or smaller further from the cutoff. Fourth, this paper only focuses on HF; additional work is needed to explore the effect of the HRRP on other targeted conditions. Finally, the HRRP could also increase the utilization of observation stays, a possibility not addressed here. An increase in the overall utilization of observation status would not necessarily lead to an increase in the duration of observation stays. These limitations represent possibilities for future research.

This paper has described the use of RD, a powerful quasi-experimental method used to evaluate interventions or policies where treatment is assigned based on a variable with a seemingly arbitrary cutoff value. Under these circumstances, RD provides an unbiased estimate of the treatment effect. Additionally, RD offers several advantages for the evaluation of complex interventions [38]. First, RD can be used in situations where randomization is not feasible or ethical. Second, RD analysis can be completed using observational data. As a result, RD is capable of answering research questions using data already routinely collected and available in electronic data warehouses and claims databases. Finally, results from RD are based on real world circumstances. Despite the advantages and utility of RD, the approach is still rarely used in health care [5]. Given the increasing amount of readily available health data, we believe researchers should consider whether RD is appropriate before turning to costlier study designs.

## Conclusion

This article demonstrates the use of RD to evaluate the impact of the HRRP on the duration of observation stays. The results provide evidence that some hospitals may extend observation stays to avoid payment penalties. Given the potential impact on patients, additional work evaluating the impact of the HRRP is warranted. Additionally, we believe the analytical method described here is underutilized in health services research. RD has many advantages over the traditional randomized experiment. We recommend other researchers consider the RD design for the evaluation of complex health interventions.
